# Fertility intentions and associated factors among female hospital employees with children: a cross-sectional study

**DOI:** 10.3389/fpsyg.2026.1929501

**Published:** 2026-09-08

**Authors:** Yanfei Ma, Bo Li, Jia Liu, Haili Long, Jianying Shang

**Affiliations:** Mianyang Central Hospital, School of Medicine, University of Electronic Science and Technology of China, Mianyang, Sichuan, China

**Keywords:** cross-sectional survey, female hospital workers, fertility intentions, fertility motivation, organizational support

## Abstract

**Objective:**

To investigate the fertility intentions of female hospital employees with children and to analyze associated factors, providing evidence for the development of targeted support policies and human-centered hospital management strategies.

**Methods:**

From July to September 2025, female hospital employees with children from hospitals in Mianyang, Sichuan Province, China, were recruited. Data were collected using a demographic questionnaire, the Fertility Motivation Scale, and the Perceived Organizational Support Scale. Statistical analyses were conducted using SPSS 25.0.

**Results:**

A total of 1,133 questionnaires were distributed, and 1,092 valid responses were returned, yielding an effective response rate of 96.38%. Among respondents, 978 (89.6%) reported no intention of having additional children, while 114 (10.4%) expressed fertility intentions. Of those with fertility intentions, 104 intended to have one more child, and 10 intended to have two. The main reasons for not intending to have additional children were: having already given birth to three children (0.41%), age-related factors (62.99%), health-related factors (55.11%), financial considerations (78.32%), work-related factors (72.70%), time constraints (63.60%), insufficient family support (46.11%), religious beliefs (1.12%), and other reasons (8.79%). Multivariable logistic regression analysis indicated that age group, number of existing children, and fertility motivation were independent predictors of fertility intention among female hospital employees with children. Compared with the younger age group, older participants were significantly less likely to have additional fertility intentions (OR = 0.307, 95% CI: 0.215–0.437, *p* < 0.001). Similarly, an increase in the number of children was associated with a significantly lower likelihood of additional fertility intention (OR = 0.158, 95% CI: 0.075–0.332, *p* < 0.001). Regarding fertility motivation, negative fertility motivation was negatively associated with fertility intention (OR = 0.952, 95% CI: 0.926–0.978, *p* < 0.001), whereas positive fertility motivation was positively associated with fertility intention (OR = 1.038, 95% CI: 1.014–1.062, *p* = 0.002). Model fit was acceptable (Hosmer–Lemeshow test *p* > 0.05) and the model explained 9.8–20.1% of variance (Nagelkerke *R*^2^ = 0.201).

**Conclusion:**

Overall, female hospital employees with children in Mianyang demonstrated a relatively low intention for additional childbearing. Fertility intention was significantly influenced by age, number of existing children, and fertility motivation, all of which were independent predictors. Future research should further explore multidimensional factors influencing fertility intentions among female healthcare workers. In addition, policy-level, organizational, and family-level interventions are needed to create a more fertility-friendly environment. Tailored strategies considering individual and family characteristics may help promote rational fertility decision-making and improve work–family balance among female healthcare professionals.

## 1 Introduction

In recent years, the continuous decline in fertility rates has become a major concern in demographic research. Over the past three decades, fertility decline has posed significant challenges to global population structure and social development (Araban et al., 2020). This decline not only affects population growth but is also closely linked to labor supply and the sustainability of social security systems, prompting many countries to implement policies to encourage childbearing (Hosseinpoor et al., 2016).

China is also facing the pressure of low fertility. Data from the Seventh National Population Census indicate that the total fertility rate in 2020 was only 1.3, placing the country in a “low fertility trap.” To address population aging and declining birth rates, China has successively implemented policies such as the “Selective Two-Child Policy,” the “Universal Two-Child Policy,” and the “Three-Child Policy” (Ren et al., 2024). Nevertheless, the overall fertility rate has not shown significant improvement (Zhou and Guo, 2020). A national survey reported that only 5.01% of women of childbearing age expressed an intention to have a third child (Yang et al., 2023). Previous studies have shown that fertility intentions are influenced by multiple socioeconomic factors, particularly economic constraints and childbearing- or childcare-related costs (Ren et al., 2024; Ren et al., 2026). Further surveys indicate that the ideal number of children for the vast majority of people of childbearing age in China is typically two, while less than 10% of women are willing to have a third child, with the proportion even lower among individuals under 30 years old (Zhang et al., 2022).

Fertility intention refers to an individual’s attitudes and expectations regarding childbearing, including the desired number of children, timing, and gender preference, and can also be understood as a psychological attitude and behavioral tendency toward reproduction (Huang et al., 2020). Previous research has shown that fertility intentions are influenced by a range of factors, including economic conditions, educational level, age, marital status, and occupational stress (Ren et al., 2024; Lee and Hwang, 2017; Yan et al., 2021; Zhu et al., 2022; Lau et al., 2018). For women considering additional children, economic factors often play a dominant role, whereas first-time mothers are more influenced by marital relationships and career planning (Yang et al., 2023). In addition, governmental childcare support policies and the sharing of parenting responsibilities between spouses significantly affect the fertility intentions of professional women (Lee and Hwang, 2017; Ranjbar et al., 2024).

Among occupational groups, female hospital employees often experience high work pressure, heavy workloads, frequent shift work, and occupational risks. Healthcare professionals, particularly nurses, differ from other female workers in terms of professional responsibilities, shift work schedules, and workplace environments, all of which may influence reproductive decision-making (Abe et al., 2025). Previous studies have investigated fertility intentions among healthcare workers and suggested that career development, work–family conflict, workplace support, and gender-related differences may influence fertility intentions (Zhang et al., 2025). However, most existing studies have focused on healthcare workers as a whole or on gender differences, while evidence specifically concerning female hospital employees with children and the factors associated with their fertility intentions remains limited. Given the unique occupational characteristics of female hospital employees, findings from studies of the general female population may not be directly applicable to this group. A previous study found that maternal and child health nurses experienced moderate levels of burnout and occupational stress (Tian, 2025). Another study found that hospital administrative staff exhibited moderate work control, high psychological job demands, moderate physical job demands, and relatively high social support, with work demands and social support identified as key factors influencing occupational burnout (Lin et al., 2025). Therefore, female hospital employees face unique challenges in balancing career development with family and childbearing responsibilities, which may further impact their fertility decisions. Seraj Shirvan et al. suggested that attention should be paid to differences in fertility intentions and behaviors among professional groups, such as career women, under fertility incentive measures (Seraj Shirvan et al., 2024). Against this background, this study focuses on female hospital employees with children in hospitals in Mianyang, Sichuan Province, China, to investigate their fertility intentions and associated factors. The aim is to provide theoretical support and practical guidance for optimizing fertility support policies for professional women and improving fertility-friendly work environments in hospitals.

## 2 Methods

### 2.1 Study design

This study was designed as a cross-sectional survey. Data were collected once from eligible female hospital employees using a self-administered questionnaire during the study period, and no follow-up was conducted.

### 2.2 Study population

From July to September 2025, female hospital employees with childbirth experience working in hospitals in Mianyang, Sichuan Province, China, were recruited using convenience sampling.

#### 2.2.1 Inclusion criteria

(1) Officially employed female staff working in the hospital; (2) Having at least one childbirth experience; (3) Willing to participate in the study and complete the questionnaire.

#### 2.2.2 Exclusion criteria

(1) Female staff who were interns, in standardized training, or undertaking advanced studies; (2) Those who had been on leave for ≥6 months in the past year; (3) Individuals with a history of psychological disorders.

#### 2.2.3 Elimination criteria

(1) Questionnaires with more than 20% missing responses; (2) Responses with obvious logical inconsistencies.

#### 2.2.4 Sample size calculation

According to the commonly recommended rule for multivariable analysis, the required sample size should be at least 10–20 times the number of independent variables included in the model (Harrell et al., 1985). A total of 21 independent variables were considered in this study. Assuming a minimum of 10 participants per variable and allowing for a 20% invalid questionnaire rate, the minimum required sample size was calculated to be 263 participants. Ultimately, 1,092 valid questionnaires were included in the analysis, which exceeded the minimum required sample size.

### 2.3 Measurement instruments

#### 2.3.1 Demographic questionnaire

Based on evidence-based medicine and nursing principles and a review of relevant domestic and international literature, the research team developed a self-designed demographic questionnaire. The questionnaire collected information on participants’ self-reported gender, age (in years), educational level, marital status, number of children, proportion of time spent on childcare, professional title, position, average number of night shifts per month, and average monthly salary. Age was collected as a continuous variable and categorized into predefined age groups for statistical analysis. The remaining demographic variables were collected as self-reported categorical variables.

#### 2.3.2 Fertility Motivation Scale

The Fertility Motivation Scale was developed by Liu et al. (2025). The original version of the scale was adopted without modification in this study. It includes 31 items, divided into two independent subscales: the Positive Fertility Motivation Subscale and the Negative Fertility Motivation Subscale. The Positive Fertility Motivation Subscale contains 18 items, covering the dimensions of desire for children and customary norms, and is used to assess individuals’ positive psychological and social motivations toward childbearing. In the original scale development study, the Positive Fertility Motivation Subscale demonstrated good internal consistency (Cronbach’s α = 0.94), and its scores were positively correlated with fertility intention. The Negative Fertility Motivation Subscale contains 13 items, covering the dimensions of emotional–social concerns and physical-material concerns, and is used to assess individuals’ concerns and negative attitudes toward childbearing. In the original study, the Negative Fertility Motivation Subscale also demonstrated good internal consistency (Cronbach’s α = 0.91), and its scores were negatively correlated with fertility intention.

Perceived Organizational Support Scale: Perceived Organizational Support Scale: The Perceived Organizational Support Scale was developed by Jahn et al. (2003) in 2003. The original version of the scale was adopted without modification in this study. It is used to assess employees’ perceptions of the extent to which their organization values their contributions and cares about their well-being. The scale consists of 9 items rated on a 5-point Likert scale ranging from strongly disagree (1) to strongly agree (5), with higher total scores indicating higher perceived organizational support. The reliability and validity of the Chinese version were verified among Chinese employees by Wang (2015), with a reported Cronbach’s α of 0.948.

### 2.4 Data collection

The study used an online survey platform (Wenjuanxing) to administer electronic questionnaires. Participants were recruited using convenience sampling. The principal investigator contacted the nursing directors of hospitals in Mianyang, Sichuan Province, China, explained the purpose of the study, and, with their assistance, distributed the questionnaire links to eligible female hospital employees with childbirth experience. The first page of the questionnaire included an informed consent statement, and participants were required to select “Agree to participate” before proceeding. The questionnaire was completed anonymously, and no personally identifiable information was collected. All responses were used solely for research purposes and were accessible only to the research team. To ensure the authenticity and independence of responses, each mobile phone number and IP address was permitted to submit only one questionnaire. The expected completion time was approximately 5–15 min. Questionnaires completed in an unusually short or long time were identified during data screening and further evaluated for eligibility. During questionnaire completion, participants could contact the principal investigator if they had any questions.

### 2.5 Quality control

All research team members involved in data collection and processing received standardized training and were familiar with the questionnaire content and completion procedures. To ensure data accuracy and completeness, two researchers independently entered all responses and cross-checked the entries. Discrepancies were resolved by referring back to the original questionnaires. Questionnaires with logical inconsistencies, completion times that were too short, or more than 20% missing data were excluded to maintain the scientific rigor and reliability of the dataset.

### 2.6 Statistical analysis

Data were entered and analyzed using SPSS version 25.0. Categorical variables were summarized as frequencies (*n*) and percentages (%). The chi-square (*χ^2^*) test was used to compare fertility intentions across different demographic and occupational characteristics, such as age, educational level, department, professional title, and number of children. When the theoretical frequency assumptions for *χ^2^* tests were not met, Fisher’s exact test was applied. A significance level of *α* = 0.05 was used.

### 2.7 Ethical considerations

This study was approved by the Ethics Committee of Mianyang Central Hospital (Approval No. S20250244-01, approved on July 11, 2025). Written informed consent was obtained from all participants prior to participation. Participants completed the questionnaire anonymously. All collected data were de-identified and used exclusively for research purposes. Access to the data was restricted to the research team to ensure participant confidentiality and privacy. All procedures performed in this study were conducted in accordance with the ethical principles of the Declaration of Helsinki.

## 3 Results

### 3.1 Demographic and occupational characteristics of female hospital employees with children

A total of 1,133 questionnaires were collected, with 1,092 valid responses, yielding an effective response rate of 96.38%. The average questionnaire completion time was 7.15 ± 5.29 min. Among the participants, 10.8% were under 30 years old, 62.5% were aged 30–39, 23.4% were 40–49, and 3.29% were over 49 years old. The majority were married (96.2%), while 3.8% were divorced or widowed. Ethnically, 96.1% were Han, and 3.9% belonged to ethnic minorities. Regarding education, 15.8% held a college diploma, 81.7% had a bachelor’s degree, and 2.5% had a master’s degree or higher. Nearly half of the participants (47.7%) were only children. In terms of hospital type, 99.5% worked in public hospitals, with 77.7% from tertiary hospitals. Regarding job categories, 80.9% were nurses, 14.3% were medical staff, 1.8% were administrative staff, and 1.7% were allied health professionals. Detailed characteristics are presented in Table 1.

**Table 1:** Demographic and occupational characteristics of 1,092 female hospital employees with children (*N* = 1,092).

Variable	Frequency	Percentage (%)	No fertility intention (*N* = 978)	Fertility Intention (*N* = 114)	*χ*^2^/Fisher	*p*
Age (years)					39.244	<0.001
<30	118	10.8	90	28		
30–39	683	62.5	606	77		
40–49	255	23.4	246	9		
>49	36	3.29	36	0		
Marital status					0165	0.685
Married	1,051	96.2	940	111		
Divorced or Widowed	41	3.8	38	3		
Ethnicity					0.591	0.442
Han	1,049	96.1	941	108		
Ethnic minorities	43	3.9	37	6		
Education level					1.979	0.377
College diploma	173	15.8	160	13		
Bachelor’s degree	892	81.7	793	99		
Master’s degree or above	27	2.5	25	2		
Only child status					1.236	0.266
No	571	52.3	517	54		
Yes	521	47.7	461	60		
Number and type of existing children					47.730	<0.001
One girl	392	35.9	329	63		
One boy	371	34	327	44		
Two boys	86	7.9	85	1		
Two girls	82	7.5	80	2		
One boy and one girl	157	14.4	154	3		
Three children	4	0.4	3	1		
History of assisted reproduction					0.435	0.510
No	1,004		901	103		
Yes	88		77	11		
Hospital level					2.490	0.115
Tertiary hospital	849	77.7	767	82		
Non-tertiary hospital	243	22.3	211	32		
Hospital type					1.000	0.576
Public hospital	1,087	99.5	973	114		
Private hospital	5	0.5	5	0		
Job category					3.301	0.472
Nursing	883	80.9	790	93		
Medical staff	156	14.3	142	14		
Administrative staff	20	1.8	17	3		
Allied health professionals	19	1.7	18	1		
Others	14	1.3	11	3		

### 3.2 Fertility status and fertility intentions of female hospital employees with children

Among the 1,092 female hospital employees with children, the current distribution of children was as follows: one girl (35.9%), one boy (34.0%), two boys (7.9%), two girls (7.5%), one boy and one girl (14.4%), and three children (0.4%). Regarding the age of the first child, 9.8% were 0–1 years old, 12.1% were 1–3 years old, 20.4% were 3–6 years old, 31.9% were 6–12 years old, 14.0% were 12–18 years old, and 11.8% were over 18 years old.

A total of 978 participants (89.6%) reported no intention to have additional children. Within this group, 9.2% were under 30 years old, 61.9% were 30–39, 25.2% were 40–49, and 3.7% were over 49. Married participants accounted for 96.1%, Han ethnicity 96.2%, and education levels were 16.4% college diploma, 81.0% bachelor’s degree, and 2.6% master’s degree or above. Nearly half (47.1%) were only children. Regarding existing children, 67.0% had one child (one girl 33.6%, one boy 33.4%), 32.6% had two children (two boys 8.7%, two girls 8.2%, one boy and one girl 15.7%), and 0.3% had three children. A history of assisted reproduction was reported by 7.9%. Most participants worked in tertiary hospitals (78.4%) and public hospitals (99.5%). Job categories included nursing (80.8%), medical (14.5%), administrative (1.7%), allied health (1.8%), and others (1.2%).

The main reasons for not intending to have more children were: already having three children (0.41%), age factors (62.99%), health factors (55.11%), economic factors (78.32%), work-related factors (72.70%), time constraints (63.60%), family support (46.11%), religious beliefs (1.12%), and other reasons (8.79%).

Among the 114 participants (10.4%) who reported an intention to have additional children, 104 (91.2%) intended to have one more child and 10 (8.8%) intended to have two more children. The willingness to have another child was 14.0% among those with one child and 1.4% among those with two children. Within this subgroup, 24.6% were under 30 years old, 67.5% were 30–39, and 7.9% were 40–49. Married participants accounted for 97.4%, Han ethnicity 94.7%, and education levels were 11.4% college diploma, 86.8% bachelor’s degree, and 1.8% master’s degree or above. More than half (52.6%) were only children. Regarding existing children, 93.9% had one child (one girl 55.3%, one boy 38.6%), 5.3% had two children (two boys 0.9%, two girls 1.8%, one boy and one girl 2.6%), and 0.8% had three children. A history of assisted reproduction was reported by 9.6%. Most participants worked in tertiary hospitals (71.9%) and public hospitals (100%). Job categories included nursing (81.6%), medical (12.3%), administrative (2.6%), allied health (0.9%), and others (2.6%).

For the 114 participants intending to have additional children, the anticipated primary caregivers for future children were distributed as follows: domestic helpers (1.8%), both parents (29.8%), paternal grandparents (31.6%), maternal grandparents (33.3%), and others (3.5%).

### 3.3 Univariate analysis of fertility intentions

Among the 1,092 female hospital employees with children, 978 (89.6%) reported no intention to have additional children, while 114 (10.4%) expressed a desire for further childbearing. Participants were divided into two groups based on fertility intention. Univariate analysis revealed that participants with fertility intention were generally younger, more likely to have only one child, and differed significantly from those without fertility intention in the number and type of existing children, as well as the scores of the Positive and Negative Fertility Motivation Subscales and their corresponding dimensions (all *p* < 0.05). No statistically significant differences were observed for the remaining demographic and occupational characteristics (all *p* > 0.05) (Tables 1, 2).

**Table 2:** Associations between fertility motivation, perceived organizational support, and fertility intentions among 1,092 female hospital employees with children (*N* = 1,092).

Variable	No fertility intention (*N* = 978)	Fertility Intention (*N* = 114)	*t*	*p*
Negative fertility motivation	25.61 ± 8.875	22.39 ± 7.84	4.091	<0.001
Emotional and social dimension	13.69 ± 5.878	11.25 ± 5.072	4.791	<0.001
Physical and material dimension	11.91 ± 3.641	11.14 ± 3.722	2.139	0.033
Positive fertility motivation	45.24 ± 10.803	48.83 ± 8.911	−3.975	<0.001
Pursuit and desire dimension	30.74 ± 6.207	32.8 ± 5.073	−4.006	<0.001
Norms and customs dimension	14.51 ± 5.793	16.04 ± 5.12	−2.972	0.003
Perceived organizational support	27.45 ± 7.53	29.39 ± 7.32	0.092	0.927

### 3.4 Multivariable logistic regression analysis of fertility intention among female hospital employees with children

A binary logistic regression model was constructed with fertility intention as the dependent variable (0 = no fertility intention, 1 = having fertility intention) to identify factors associated with the likelihood of additional childbearing. The independent variables were coded as follows: age was entered as a categorical variable (with the youngest age group as the reference category); number of existing children was coded as 1, 2, and ≥3 children, with one child as the reference group; positive and negative fertility motivation were included as continuous variables using total scale scores. The Enter method was applied for multivariable analysis.

The multivariable logistic regression analysis identified age group, number of existing children, and fertility motivation as independent factors associated with fertility intention.

Compared with the younger age group, participants in the older age group were significantly less likely to have additional fertility intentions (OR = 0.307, 95% CI: 0.215–0.437, *p* < 0.001).

Similarly, an increased number of existing children was significantly associated with a lower likelihood of fertility intention (OR = 0.158, 95% CI: 0.075–0.332, *p* < 0.001), indicating that women with more existing children were less likely to intend further childbearing.

Regarding fertility motivation, negative fertility motivation was negatively associated with fertility intention (OR = 0.952, 95% CI: 0.926–0.978, *p* < 0.001), whereas positive fertility motivation was positively associated with fertility intention (OR = 1.038, 95% CI: 1.014–1.062, *p* = 0.002). These findings indicate that both positive and negative fertility motivation were independently associated with fertility intention.

Model fit assessment indicated a good goodness-of-fit (Hosmer–Lemeshow test: χ^2^ = 3.646, *p* = 0.888). The overall model was statistically significant (Omnibus test: χ^2^ = 112.526, *p* < 0.001), with moderate explanatory power (Cox and Snell *R*^2^ = 0.098; Nagelkerke *R*^2^ = 0.201). The overall classification accuracy was 89.7%. However, the model showed relatively low sensitivity in identifying participants with fertility intention, suggesting that class imbalance may have affected predictive performance. The detailed multivariable logistic regression results are presented in Table 3.

**Table 3:** Multivariable logistic regression analysis of fertility intention among female hospital employees with children (*N* = 1,092).

Variable	B	SE	*p*	OR	95% CI (lower)	95% CI (upper)
Age	−1.181	0.181	<0.001	0.307	0.215	0.437
Number of children	−1.848	0.380	<0.001	0.158	0.075	0.332
Negative fertility motivation	−0.049	0.014	<0.001	0.952	0.926	0.978
Positive fertility motivation	0.037	0.012	0.002	1.038	1.014	1.062

## 4 Discussion

Population and fertility issues represent complex, multidimensional challenges in human societies. Fertility rate, as a critical determinant of population growth, is closely linked to the sustainable development of social and economic systems. Consequently, many countries have developed and implemented various incentive policies to address the challenges posed by declining fertility rates (Li et al., 2019). When formulating such policies, it is essential to consider socioeconomic and cultural contexts to implement more targeted measures and effectively enhance fertility among reproductive-age populations (Ranjbar et al., 2025; Dong et al., 2024). However, increasing the desired number of children among married individuals remains a global social challenge (Atake and Gnakou Ali, 2019).

In the present study, the majority of female hospital employees with children in Mianyang were aged 30–39 years (62.5%), held a bachelor’s degree or higher, and were primarily employed in nursing positions (80.9%). Most participants were married, long-term employees of public tertiary hospitals, indicating a group with relatively high educational attainment and occupational stability. Given the fast-paced and high-pressure nature of the healthcare profession, these women may face challenges in balancing childbearing and career development. Notably, nearly half of the respondents (47.7%) were only children. Individuals from only-child families may bear greater responsibilities for parental support and childcare within their family structures, and the absence of siblings to share these responsibilities may contribute to psychological burdens affecting decisions about additional childbearing. This finding suggests that, in the context of an underdeveloped social support system, limited familial support resources may serve as a significant constraint on women’s willingness to have more children. Previous studies have indicated that only-child family structures not only influence intergenerational care and parent–child relationships but also have long-term implications for population structure, which increasingly attracts the attention of policymakers (Seraj Shirvan et al., 2024). These observations highlight the potential benefits of enhancing social and familial support systems, particularly measures targeting only-child families, to alleviate both psychological and practical pressures on women considering further childbearing.

Seraj Shirvan et al. (2024) suggested that fertility-promoting policies should not only focus on fostering a supportive cultural environment but also be combined with economic incentives, with particular attention to the specific needs of professional women and those with higher education. This study focused on female hospital employees with children, and the results indicated that the overall intention for additional childbearing was low, with only 10.4% of participants planning to have another child. This finding reflects a relatively weak propensity for further fertility within this population, consistent with the declining fertility intentions observed among women nationwide, and lower than reported in previous studies (Yang et al., 2024; Jing et al., 2022). Among participants without fertility intentions, economic concerns (78.32%) and work-related factors (72.70%) were reported as the primary deterrents, followed by time constraints (63.60%), age (62.99%), and physical health (55.11%). These findings align with current literature, which highlights that policy support, economic resources, ideological guidance, and income-related factors are critical determinants of fertility decisions and childbearing preferences (Yang et al., 2024). The results suggest that economic pressures and occupational workload may be key situational factors affecting the willingness of hospital female employees to have additional children. Due to the high intensity, rigid scheduling, and potential conflicts between maternity leave and career advancement in healthcare positions, female hospital employees may adopt more cautious approaches in fertility decision-making. Furthermore, insufficient family support (46.11%) may also undermine women’s confidence in childbearing, indicating that unequal distribution of childcare responsibilities within families remains a practical challenge.

Notably, among the 114 participants with fertility intentions, the vast majority (91.2%) planned to have only one additional child, while only 8.8% intended to have two, which is lower than the proportion reported by Yan et al., where 12.2% expressed willingness for a third child (Yan et al., 2021). This suggests that although some women still consider further childbearing, their plans are relatively conservative, likely reflecting rational evaluations of economic burden and caregiving demands. Collectively, these data indicate that fertility intentions among professional women tend to be characterized by “low quantity and rationalized planning.”

This study, based on multivariable logistic regression analysis, demonstrated that fertility intention among female hospital employees with children was jointly influenced by age, number of existing children, and fertility motivation, all of which were independent predictors. Age was identified as an important demographic factor associated with fertility intention. The present study found that participants in the higher age group were significantly less likely to have additional fertility intentions, which is consistent with previous findings (Chua et al., 2020). With increasing age, female fertility capacity gradually declines, while physiological risks increase and occupational stability becomes stronger, all of which may contribute to a reduced willingness for further childbearing. The number of existing children was significantly negatively associated with fertility intention. As parity increased, the likelihood of additional childbearing intention decreased markedly, suggesting that family size and childcare burden may play important roles in fertility decision-making. This finding reflects a clear “cumulative inhibitory effect” of childbirth experience, whereby a greater number of existing children imposes increased economic, temporal, and caregiving pressures on families, thereby reducing the likelihood of further fertility. In addition, Ning et al. (2022) reported that the gender composition of the first two children is an important determinant of subsequent childbearing decisions. At the psychological level, fertility motivation was found to significantly influence fertility intention. Positive fertility motivation promoted the intention for additional childbearing, whereas negative fertility motivation exerted an inhibitory effect. These findings indicate that fertility decision-making among women is not only shaped by objective demographic factors but is also influenced by psychological cognition and emotional attitudes, highlighting the complexity and multidimensional nature of reproductive decision-making. Interestingly, perceived organizational support was not identified as an independent predictor of fertility intention in the multivariable analysis. One possible explanation is that the effect of perceived organizational support on fertility intention may be indirect and may be overshadowed by more proximal psychological and family-related factors. Previous studies have shown that work–family conflict, fertility attitudes, and child-rearing burden are important determinants of women’s fertility intention (Li et al., 2024). Therefore, after adjustment for demographic characteristics and fertility motivation, the independent association between perceived organizational support and fertility intention may have been attenuated. In addition, the relatively limited variability in perceived organizational support among participants may also have reduced its statistical association with fertility intention. Future studies are warranted to further explore the potential mechanisms through which organizational support may influence fertility intention.

In summary, fertility intention among female hospital employees with children appears to be associated with a combination of demographic characteristics, including age and number of existing children, as well as psychological factors such as fertility motivation. These findings suggest that future efforts to improve fertility intention among female hospital employees should take these factors into consideration while fostering a supportive environment that facilitates work–family balance. Further research is needed to evaluate the effectiveness of such approaches in different healthcare settings.

### 4.1 Limitations

This study focused on female hospital employees with children in Mianyang and adopted a cross-sectional study design. Therefore, causal inferences between variables cannot be established. In addition, although multivariable logistic regression analysis was conducted, the inherent limitations of cross-sectional data still restrict the ability to infer temporal or causal relationships among age, fertility motivation, number of children, and fertility intention. Moreover, several potentially important confounding factors, such as household income, spouse’s socioeconomic status, employment contract type, history of infertility, and maternity leave policies, were not collected in the present study and therefore could not be adjusted for in the analysis. These unmeasured factors may have influenced fertility intention and should be considered in future studies.

Furthermore, the study sample was primarily drawn from hospitals in the Mianyang region, which may limit the generalizability of the findings to female healthcare workers in other regions or healthcare settings. Future research should recruit more geographically diverse samples, incorporate additional sociodemographic and occupational variables, and adopt longitudinal or mixed-methods designs to further explore the dynamic changes and underlying mechanisms of fertility intention among female healthcare professionals. In addition, intervention studies are warranted to evaluate the effectiveness of targeted organizational and social support strategies in promoting fertility intention and improving work–family balance.

## 5 Conclusion

The overall intention for additional childbearing among female hospital employees with children was low, with only a small proportion planning to have another child. Economic burden, occupational stress, insufficient time, and lack of family support were identified as the primary influencing factors. Age, the number and gender composition of existing children, as well as positive and negative fertility motivations, all significantly affected the intention for further childbearing. Fertility decisions among professional women exhibited a pattern of “low quantity and rationalized planning.” To enhance fertility intentions in this population, it is recommended to improve family-friendly policies at the policy level, strengthen family and community support systems at the societal level, and optimize the work environment and career advancement mechanisms at the organizational level, thereby enabling women to better balance career development with family responsibilities.

## Data Availability

The original contributions presented in the study are included in the article/supplementary material, further inquiries can be directed to the corresponding authors.
